# An activation specific anti-Mac-1 designed ankyrin repeat protein improves survival in a mouse model of acute lung injury

**DOI:** 10.1038/s41598-022-10090-6

**Published:** 2022-04-15

**Authors:** Patrick M. Siegel, Anne-Sophie Przewosnik, Jan Wrobel, Timo Heidt, Martin Moser, Karlheinz Peter, Christoph Bode, Philipp Diehl, István Bojti

**Affiliations:** 1 Department of Cardiology and Angiology I, University Heart Center Freiburg – Bad Krozingen, Faculty of Medicine, University of Freiburg, Hugstetter Str. 55, 79106 Freiburg, Germany; 2 Atherothrombosis and Vascular Biology Laboratory, Baker Heart and Diabetes Institute, Melbourne, Australia; 3 Department of Medicine, Central Clinical School, Monash University, Melbourne, Australia; 4 Department of Cardiometabolic Health, University of Melbourne, Melbourne, Australia

**Keywords:** Immunology, Molecular biology, Diseases, Medical research, Molecular medicine

## Abstract

The acute respiratory distress syndrome (ARDS) is a life-threatening clinical condition. The number of ARDS cases has risen dramatically recently but specific treatment options are limited. ARDS is associated with an overshooting inflammatory response and neutrophils play a central role in its pathogenesis. Neutrophils express the integrin Mac-1 on their surface which adopts a resting and activated conformation depending on leukocyte activation. The aim of this study was to investigate the anti-inflammatory effects of the unique activation-specific anti-Mac-1 DARPin ‘F7’ in a mouse model of ARDS. ARDS was induced by intratracheal lipopolysaccharide (LPS) instillation and the acute (day 1–4) and chronic phase (day 5–10) were studied. After expression and purification, F7, a control DARPin and PBS, were applied daily via the intraperitoneal route. Survival and weight loss were recorded. Histological analysis of lung sections, flow cytometric leukocyte analysis of blood and bronchioalveolar lavage (BALF) were performed. Moreover, protein concentration and cytokine levels were determined in the BALF. Treatment with F7 improved survival and reduced weight loss significantly compared to treatment with the control DARPin or PBS. Neutrophil count in the BALF and peripheral blood were significantly reduced in mice treated with F7. Histology revealed significantly reduced pulmonary inflammation in the F7 treated group. Treatment with DARPin F7 inhibited neutrophil accumulation, reduced signs of local and systemic inflammation and improved survival in a mouse model of ARDS. F7 may be a novel anti-inflammatory drug candidate for the treatment of severe ARDS.

## Introduction

The acute respiratory distress syndrome (ARDS) is a life threatening clinical condition characterized by severe acute hypoxemia and bilateral radiological opacities, which develop within one week and are not fully explained by cardiac failure or fluid overload^1,2^. ARDS may be triggered by several causes, the most common being trauma, aspiration, extrapulmonary sepsis or pneumonia^1,3^. ARDS has moved to the center of public and scientific attention due to the ongoing SARS-CoV-2 pandemic.

Mortality due to ARDS remains high as therapeutic options are limited to the treatment of the underlying condition, if possible, and supportive therapy including, fluid management (diuretics) mechanical ventilation and extracorporeal membrane oxygenation^2,4–6^. Consequently, there is an urgent need for novel therapeutic strategies^7^.

Neutrophils play a central role in the pathogenesis of ARDS^8^. The leukocyte integrin Mac-1, which is highly expressed on neutrophils, is crucial for leukocyte migration through the endothelium rendering it an attractive therapeutic target^9^. Mac-1 adopts a resting and activated conformation, both with distinct functions^10^.

Designed Ankyrin Repeat Proteins (DARPins) are a novel class of binding proteins with multiple advantages over antibody drugs^11^. Compared to conventional immunoglobulins, DARPins bind to their targets with similar or even higher affinity but offer several advantages such as higher stability and cost-effective production^12,13^. We recently assessed the anti-inflammatory effects of a *unique* activation-specific anti-Mac-1 DARPin ‘F7’ in mouse models of myocarditis, sepsis and myocardial infarction^14^.

A realistic and highly reproducible mouse model of the acute lung injury (ALI) is the intrapulmonary lipopolysaccharide (LPS) application^15^. Intratracheal administration of LPS leads to reliable neutrophil accumulation, alveolar wall thickening, protein-rich edema and detritus in the alveolar space^16^.

The aim of this study was to find a novel anti-inflammatory treatment option for ARDS. For this purpose, the therapeutic effects of the activation-specific anti-Mac-1 DARPin ‘F7’ were assessed in a mouse ARDS model of intrapulmonary LPS application.

## Results

**Figure 1:**
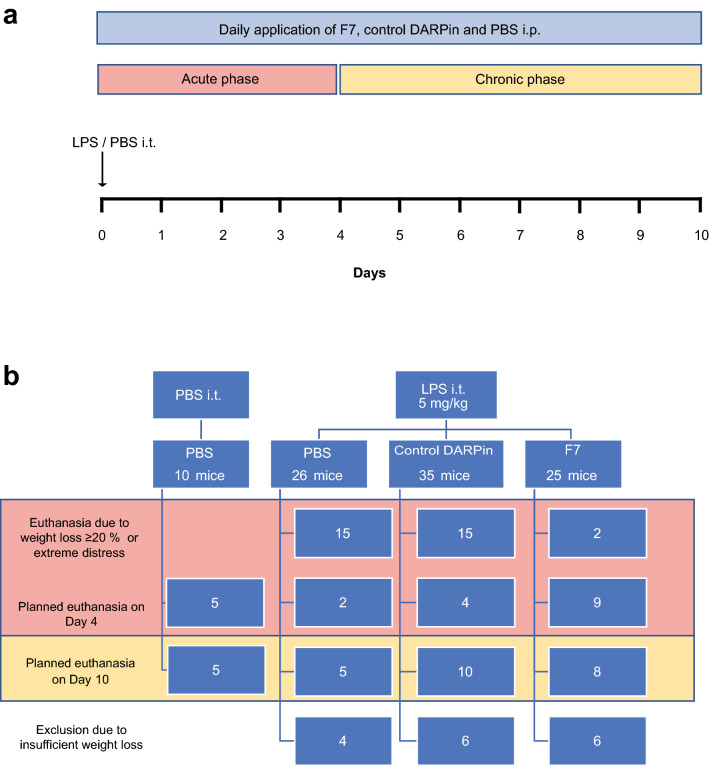
Conceptual figure describing the mouse model of LPS-induced acute lung injury and treatments. (**a**) Mice received 200 µg of F7, control DARPin and PBS daily via the intraperitoneal (i.p.) route beginning on day 0. On day 0 mice received intratracheal (i.t.) LPS (for F7, control DARPin and PBS group) or intratracheal PBS (for healthy controls). Day 1 to day 4 was considered the acute phase. Day 4–10 was considered the chronic phase of acute lung injury. (**b**) Overview of the number of mice in the different treatment groups and the time point of euthanasia. The number of mice per treatment group was determined according to the expected survival rate and the number of mice needed to show significant differences. Mice were pre-assigned to the acute or chronic phase. Planned euthanasia was conducted on day 4 (acute phase) or day 10 (chronic phase). Mice were euthanized early if they showed signs of severe weight loss or distress (Table 1) and were then counted as non-survivors. Several mice in the PBS and control DARPin group which were pre-assigned to the chronic phase had to be sacrificed early as they reached criteria for euthanasia.

The experimental protocols were approved by the responsible local authorities (‘Regierungspräsidium Freiburg’, 35-9185.81/G-19/122). Only male mice were used. The mouse model of acute lung injury consists of the acute and chronic phase (Fig. 1). Four treatment groups were formed (healthy controls, PBS, control DARPin, F7). Mice within a treatment group were pre-assigned to the ‘acute’ (day 1–4) or ‘chronic’ (day 5–10) phase before application of LPS (No. of mice in the acute phase: healthy controls: 5, control DARPin: 19, F7: 11, PBS: 17). To comply with our animal ethics approval, the lowest number of mice per treatment group and phase needed to show significant differences was chosen taking into account the expected survival rate in the literature^17^. Several mice in each treatment group (and particularly the control groups) including mice pre-assigned to the chronic phase had to be euthanized early as they reached criteria for early euthanasia (Table 1). These mice were then switched to the acute phase. On day 4, the remaining mice originally assigned to the acute phase, that had not suffered extreme weight loss were euthanized. The remaining mice in each group were euthanized on day 10 and were part of the ‘chronic phase’. 4–6 mice in each treatment group had to be excluded from analysis post-hoc as they did not develop weight loss at all and application of LPS had most likely been intragastric.

**Table 1:** Criteria for euthanasia.

Metric criteria	Weight loss ≥ 20% compared to the start weight (100%)
Parametric criteria	Tachy-/bradypnea with use of accessory breathing muscles (90.6%)
	Severe disturbance in coordination (12.5%)
	Paralysis of the extremities (0%)
	Autoaggressivity (0%)
	Coma (0%)

According to the Society of Laboratory Animal Science^47^, the following criteria were controlled on every day of the experiment. If one of them was fulfilled, the mouse was euthanized. (occurrence is signalized as percentage of all prematurely euthanized animals).

### Acute phase

The first 4 days after endotracheal application of LPS were considered the acute phase of acute lung injury. 15 of the PBS treated, 2 of the F7 treated and 15 of the control DARPin treated animals reached the criteria for euthanasia (as defined in the “Methods” section) and were euthanized early (on day 2 or 3). A total of 17 mice in the PBS group, 19 mice in the control DARPin group and 11 mice in the F7 group were counted towards the acute phase and blood, BALF and lung tissue were analyzed. All healthy animals (no LPS treatment) survived.

### DARPin F7 improves survival

**Figure 2:**
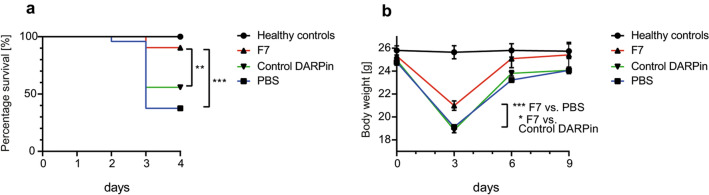
Treatment with F7 improves survival and reduces weight loss during the acute phase in a mouse model of acute lung injury. (**a**) Kaplan–Meier plot indicating percentage of survival on different days during the acute phase. Terminally ill animals were euthanized according to the study protocol (Table 1). Probability of survival was calculated by dividing the number of surviving mice by the number of total mice (acute and chronic phase) and multiplying the result with 100%. The survival probability was significantly increased for mice treated with F7 compared to the PBS or the control DARPin groups. Data are presented as mean ± SEM. n = 10–35 per group, **p < 0.01, ***p < 0.001. Comparisons of overall survival were performed using the log-rank test. (**b**) Mean weight over time during the acute and chronic phase of the mouse model of acute lung injury. Mice treated with the control DARPin or PBS suffered more severe weight loss compared to mice treated with F7, particularly during the acute phase, as exemplified on day 3. Data are presented as mean ± SEM. n = 10–35 mice per treatment group, *p < 0.05, ***p < 0.001, Body weight on day 3 was compared using an unpaired t-test.

As described in the “Methods” section, animals were euthanized if they developed weight loss over 20% or if they exhibited signs of massive distress. Mice that survived until day 4 were considered as survivors. Survival was improved in the group treated with F7 (Fig. 2), whereas the probability of survival in the control groups was reduced (F7 vs. PBS vs. control DARPin (%): 90.0 vs. 30.4 vs 54.5, *p* < 0.001 for F7 vs. PBS, *p* = 0.008 for F7 vs. control DARPin).

### DARPin F7 prevents severe weight loss in the acute phase

The initial weight was similar in all groups (healthy control: 25.8 ± 0.41 g, F7: 25.31 ± 0.41 g, control DARPin: 24.96 ± 0.23 g, PBS: 24.78 ± 0.27 g). As shown in Fig. 2, significant weight loss was observed in the LPS challenged groups on day 4. Treatment with DARPin F7 significantly reduced weight loss (F7 vs. PBS vs. control DARPin (%): 16.4 ± 1.3 vs. 22.8 ± 0.64 vs. 20 ± 0.99, p < 0.001 for F7 vs PBS, *p* = 0.029 for F7 vs. control DARPin). As expected, weight loss, expressed in percentage of starting weight on day 0 was also lower in the F7 treated group (Supplementary Figure S1).

### DARPin F7 protects from a systemic inflammatory reaction in peripheral blood

**Figure 3:**
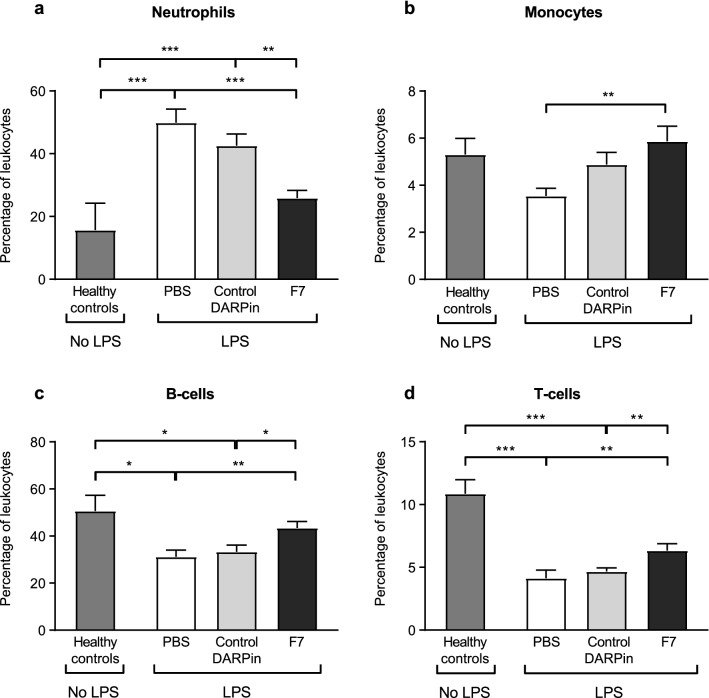
Flow cytometric analysis of blood leukocytes during the acute phase. Blood was harvested and leukocyte staining for flow cytometry was performed as described in the “Methods” section. The percentage of all leukocytes is presented. (**a**) The percentage of blood neutrophils was lower in the group treated with F7 compared to PBS or the control DARPin. (**b**) The percentage of monocytes was increased in the group treated with F7. (**c**) The percentage of B-cells was also increased in the group treated with F7 compared to PBS and control DARPin and similar to the healthy controls. (**d**) A similar observation was made for the percentage of T-cells in blood. Data are presented as mean ± SEM. n = 5–19, *p < 0.05, **p < 0.01, ***p < 0.001. Treatment groups were compared by an unpaired t-test.

As expected, the percentage of neutrophils (CD45^+^CD11b^+^Ly6C^+^Ly6G^+^) in blood was significantly increased in the group treated with PBS or the control DARPin. This effect was significantly reduced in the F7 and the healthy control group (percentage of neutrophils PBS vs. control DARPin vs. F7 vs. healthy controls: 49.9 ± 4.35 vs. 42.6 ± 3.69 vs. 25.9 ± 2.45 vs. 15.7 ± 8.54, *p* < 0.001 F7 vs. PBS, *p* = 0.002 F7 vs. control DARPin, Fig. 3). The percentage of monocytes (CD45^+^CD11b^+^Ly6C^+^Ly6G^-^) in the peripheral blood was less affected since healthy control mice, mice treated with the control DARPin and F7 did not significantly differ. A significantly reduced percentage of monocytes was observed in the PBS group compared to F7 (percentage of monocytes PBS vs F7: 3.5 ± 0.33 vs. 5.9 ± 0.65, *p* = 0.002, Fig. 3).

The percentage of B-lymphocytes (CD45^+^CD19^+^) in peripheral blood was lower in the groups treated with PBS or the control DARPin compared to F7 (percentage of B-lymphocytes PBS vs. control DARPin vs. F7: 31.13 ± 2.92 vs. 33.3 ± 2.89 vs. 43.4% ± 2.73; *p* = 0.005 for F7 vs. PBS, *p* = 0.023 for F7 vs. control DARPin). The proportion of T-lymphocytes (CD45^+^CD3^+^) was also significantly reduced in the groups treated with control DARPin or PBS compared to F7 (percentage of T-lymphocytes PBS vs. control DARPin vs. F7 vs. healthy control, 4.1 ± 0.65 vs. 4.7 ± 0.3 vs. 6.3 ± 0.55 vs. 10.9 ± 1.13, *p* = 0.005 F7 vs. PBS, *p* = 0.009 F7 vs. control DARPin).

### DARPin F7 limits the severity of neutrophil infiltration in the alveolar space in the acute phase

**Figure 4:**
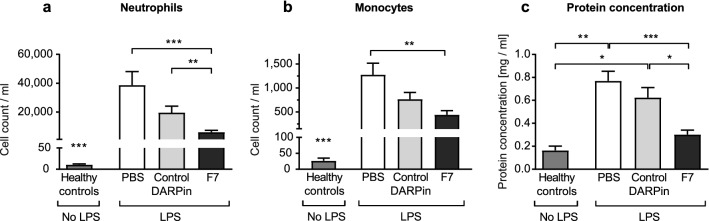
F7 reduces monocyte and neutrophil count and protein content in the bronchoalveolar lavage fluid (BALF) during the acute phase. Neutrophils and monocytes were analyzed by flow cytometry as described in the “Methods” section. Protein content in the BALF was determined using a BCA assay. Absolute cell counts were determined using Trucount beads following the manufacturer’s instructions. (**a**) Animals treated with DARPin F7 had significantly lower neutrophil counts in the BALF than the PBS or control DARPin treated groups. Compared to healthy control animals, neutrophil counts were elevated in all treatment groups. (**b**) Monocyte counts in the BALF were also significantly reduced in the F7 treated group compared to the PBS treated group. Again, monocyte counts were elevated in all treatment groups compared to healthy controls. (**c**) The protein concentration in the BALF was significantly higher in mice receiving PBS or the control DARPin compared to mice treated with F7. Data are presented as mean ± SEM. n = 5–19, *p < 0.05, **p < 0.01, ***p < 0.001. Treatment groups were compared by an unpaired t-test.

LPS induced a severe local inflammatory reaction indicated by increased absolute neutrophil counts in the BALF compared to healthy controls. However, treatment with F7 significantly reduced neutrophil influx in the bronchoalveolar lavage compared to PBS and control DARPin (neutrophil count/ml healthy control vs. PBS vs. control DARPin vs. F7: 10.4 ± 2.16 vs. 38,621 ± 9,448 vs. 19,638 ± 4,497 vs. 6,758 ± 1,345, *p* < 0.001 for F7 vs. PBS, *p* = 0.01 for F7 vs. control DARPin, Fig. 4). The level of monocytes (CD45^+^CD11b^+^Ly6C^+^Ly6G^−^) in the BALF were significantly reduced in the group treated with F7 compared to the PBS group (monocyte count/ml PBS vs. F7: 1276 ± 240.3 vs. 441 ± 87.33 *p* = 0.008). As previously described, levels of B-Lymphocytes and T-lymphocytes in the BALF were extremely low and are therefore not presented^17^.

### DARPin F7 prevents the elevation of protein content in the BALF

The protein content in the BALF can be used as a marker of pulmonary inflammation and was therefore determined. Protein content of the BALF was significantly elevated in the groups treated with PBS and the control DARPin compared healthy control animals or those treated with F7 (Protein concentration in mg/dl PBS vs. control DARPin vs. healthy controls vs. F7: 0.77 ± 0.08 vs. 0.62 ± 0.09 vs. 0.16 ± 0.04 vs. 0.3 ± 0–04, *p* < 0.001 for F7 vs PBS, *p* = 0.011 F7 vs. control DARPin; Fig. 4).

### DARPin F7 prevents histological changes in the acute phase

**Figure 5:**
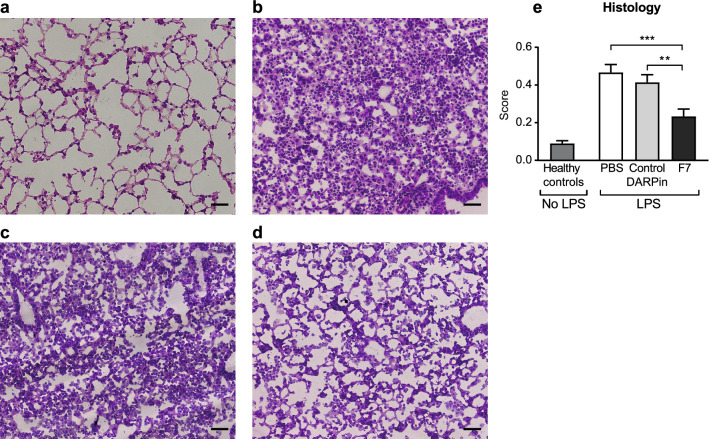
Histological analysis of lung sections shows reduction of inflammation during the acute phase. Representative histological sections of mice in the different treatment groups stained with hematoxylin–eosin: (**a**) Healthy controls, (**b**) PBS, (**c**) control DARPin, (**d**) F7. The scale bar indicates 50 µm. (**e**) The lung injury scoring system according to Matute Bello et al. was used for histological analysis and is described in the “Methods” section. The histological score of the group treated with F7 was significantly lower compared to the PBS or control DARPin treated group indicating reduced histological signs of inflammation. Data are presented as mean ± SEM. n = 5–19, **p < 0.01, ***p < 0.001. Treatment groups were compared by an unpaired t-test. The scale bar represents 50 µm.

Analysis on day 4 showed a significant elevated histological score in all groups receiving intratracheal LPS compared to the healthy control group. However, animals treated with F7 had a significantly lower histological score compared to those treated with PBS or the control DARPin (histological score PBS vs. F7 vs. control DARPin: 0.47 ± 0.042 vs. 0.23 ± 0.04 vs. 0.41 ± 0.04, *p* < 0.001 for F7 vs. PBS, *p* = 0.004 for F7 vs. control DARPin, Fig. 5). Low power whole lunge images of the different treatment groups during the acute phase are provided as Supplementary Figure S2.

### DARPin F7 reduces the IL-6 and IL-10 mRNA expression in the lung

mRNA-analysis using qPCR on day 4 showed a significant elevation of IL-6 and IL-10 expression levels in all groups compared to the healthy controls (Supplementary Figure S3). There was a significant reduction of the IL-6 and IL-10 expression in the lung tissue of mice treated with F7 compared to PBS and a marked reduction in the IL-6 and IL-10 expression in the lung tissue of mice treated with F7 compared to control DARPin (mRNA expression fold change: IL-10: PBS vs. F7 vs. control DARPin: 978.3 ± 167 vs. 218.6 ± 81.28 vs. 388.9 ± 93.1, *p* < 0.001 for F7 vs. PBS, *p* = 0.11 for F7 vs. control DARPin, *p* = 0.01 for PBS vs. control DARPin; IL-6: PBS vs. F7 vs control DARPin: 12.43 ± 3.1 vs. 3.78 ± 0.72 vs. 7.16 ± 1.43, *p* = 0.002 for F7 vs. PBS, *p* = 0.078 for F7 vs. control DARPin).

### DARPin F7 decreases cytokine levels in the BALF

Cytokine concentrations were measured in the BALF using a flow-cytometric bead array. Levels of IL-1β, TNF-α, IFN-γ, IL-6, MCP-1 and IL-12 were significantly lower in the group treated with F7 (Supplementary Figure S4) compared to mice treated with the control DARPin or PBS (e.g., concentration in pg / ml PBS vs. control DARPin vs. F7: IL-6: 729.3 ± 187.4 vs. 517.7 ± 169.6 vs. 57.97 ± 35.29, *p* = 0.002 F7 vs. PBS, *p* = 0.004 F7 vs. control DARPin; TNF-α: 138.6 ± 27.93 vs. 89.34 ± 20.10 vs. 27.28 ± 7.98, *p* = 0.016 for F7 vs. PBS, *p* = 0.005 for F7 vs. control DARPin). Cytokine levels of healthy control mice were below the limit of determination.

### Chronic phase of acute lung injury

The chronic phase of ALI was investigated in animals that survived until day 10 after LPS introduction. 5 healthy control animals, 5 mice receiving PBS, 8 treated with F7 and 10 control DARPin treated animals were analyzed.

### Body weight and peripheral blood leukocyte counts are similar in all treatment groups

**Figure 6:**
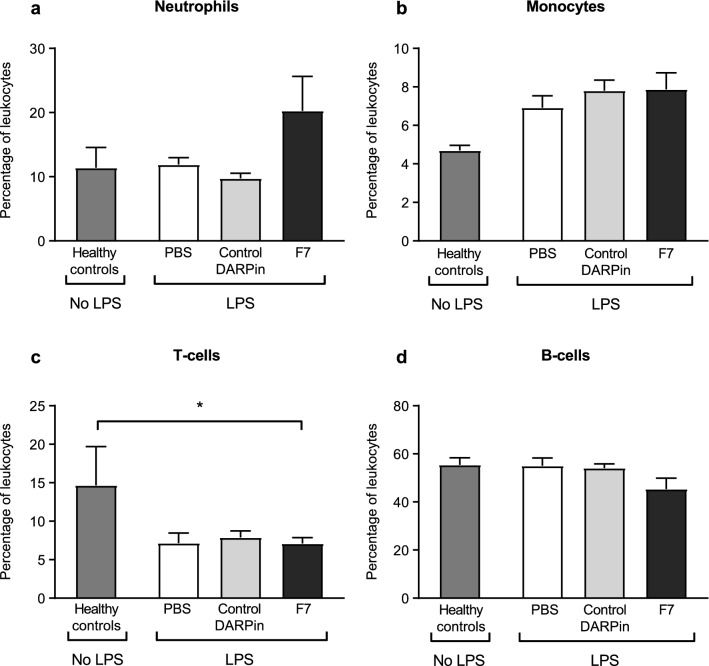
Percentages of blood leukocytes are similar between the different treatment groups in the chronic phase. Blood was harvested on day 10 and analyzed using flow cytometry as described in the “Methods” section. (**a,b**) Percentages of neutrophils and monocytes were similar between the different treatment groups. (**c**) Percentages of T-cells were significantly higher in the healthy control group compared to the F7 treated group. There was no significant difference between the other treatment groups. (**d**) No significant differences were observed regarding the percentage of B-cells between the different treatment groups and compared to healthy controls. Data are presented as mean ± SEM. n = 5–10, *p < 0.01. Treatment groups were compared by an unpaired t-test.

Animal weight was similar in all treatment groups during the chronic phase. Moreover, flow cytometric analysis revealed that peripheral leukocyte blood counts did not differ significantly between the F7, control DARPin or PBS treated groups (Fig. 6).

### DARPin F7 protects from a chronic inflammatory neutrophil accumulation in the BALF

**Figure 7:**
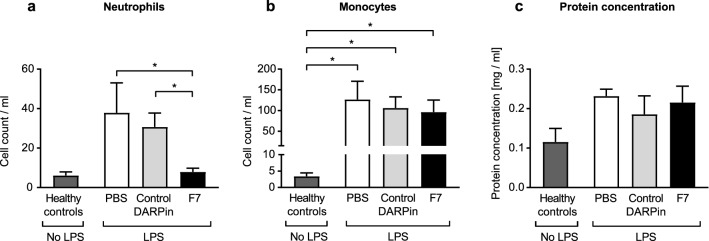
Analysis of bronchoalveolar lavage fluid (BALF) during the chronic phase reveals lower levels of neutrophils in mice treated with F7. BALF was harvested on day 10. (**a**) Neutrophil counts on day 10 were similar in healthy control animals compared to mice treated with F7, meanwhile neutrophil counts in both control groups remained significantly elevated. (**b**) Monocyte counts in the BALF on day 10 of the chronic phase did not differ significantly between treatment groups but were elevated compared to healthy control animals. (**c**) The protein concentration on day 10 of the chronic phase did not differ significantly between the different treatment groups. Data are presented as mean ± SEM. n = 5–10, *p < 0.01. Treatment groups were compared by an unpaired t-test.

While the neutrophil (CD45^+^CD11b^+^Ly6C^+^Ly6G^+^) count in the BALF of the group treated with F7 on day 10 returned to the level of the healthy control group, the neutrophil count of the PBS- and control DARPin-treated groups remained significantly elevated (neutrophil count/ml healthy controls vs. F7 vs. PBS vs control DARPin: 6.1 ± 1.78 vs. 9.02 ± 1.97 vs 37.8 ± 15.18 vs. 25.57 ± 6.67, *p* = 0.02 for F7 vs. PBS, *p* = 0.02 for F7 vs. control DARPin). The monocyte (CD45^+^CD11b^+^Ly6C^+^Ly6G^-^) count was significantly elevated in all groups receiving intratracheal LPS compared to healthy controls but showed no remaining differences between treatments (monocyte count/ml PBS vs. F7 vs. control DARPin vs. healthy controls: 126.8 ± 44.02 vs. 96.2 ± 29.06 vs. 106.4 ± 26.61 vs. 3.4 ± 1.02, *p* = 0.02 for healthy controls vs. PBS, F7 and control DARPin) (Fig. 7).

### BALF protein content and histology is similar in all treatment groups in the chronic phase

**Figure 8:**
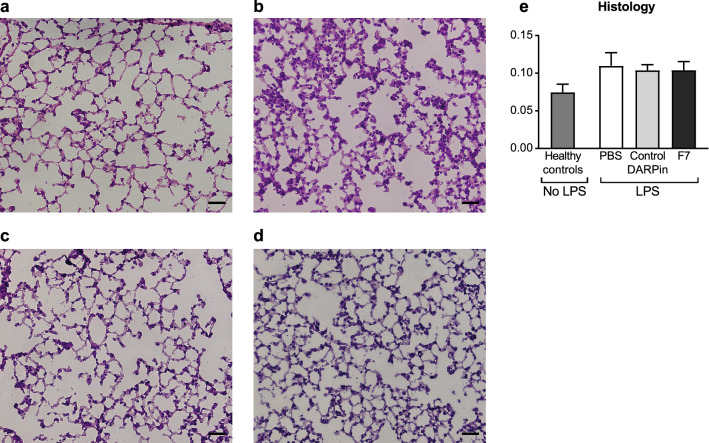
Histological analysis of lung sections during the chronic phase on day 10 reveals no significant differences between treatment groups. Representative histological sections of mice in the different treatment groups stained with hematoxylin–eosin: (**a**) Healthy controls, (**b**) PBS, (**c**) control DARPin, (**d**) F7. The scale bar indicates 50 µm. (**e**) The lung injury scoring system according to Matute Bello et al. was used for histological analysis and is described in the “Methods” section. The histological score of the group treated with F7 was not significantly different from the PBS or control DARPin treated group indicating reduced histological signs of inflammation. Data are presented as mean ± SEM. n = 5–10. Treatment groups were compared by an unpaired t-test. The scale bar represents 50 µm.

All groups receiving intratracheal LPS had similar total protein levels in the BALF on day 10 with no significant differences between treatment groups (Fig. 7). Furthermore, there were no significant differences in the histological scores between treatment groups on day 10 (Fig. 8).

## Discussion

In this study we demonstrate that the anti-Mac-1 DARPin F7 significantly inhibits the local and systemic inflammatory reaction and improves survival in a mouse model of ARDS.

ARDS is associated with high mortality rates and treatment is challenging and prolonged. A therapeutic agent, which inhibits or mitigates the development and course of the disease is urgently required.

We demonstrate that treatment with DARPin F7 reduces neutrophil infiltration into the alveoli and the percentage of neutrophils in the peripheral blood. This finding is important as neutrophils play a central role in the initiation and progression of ARDS. For example, Abraham et al. describes, that the activation and accumulation of neutrophils in the lungs is one of the first steps in the development of ARDS^18^. Moreover, there are only few cases of ARDS in patients with neutropenia^19^. Additionally, multiple studies demonstrated, that lung infiltrating neutrophils are the main cell type in the BALF in patients with ARDS^20–22^. ARDS is characterized by the injury of the alveolar endothelium (pulmonary ARDS) or the microvascular endothelium (extrapulmonary ARDS), leading to a severe inflammation of lung tissue. Irrespective of the origin, this results in interstitial and alveolar edema and a mainly neutrophil driven elevation of inflammatory mediators^2^.

We also demonstrated, that treatment with F7 reduces the intrapulmonary expression of the cytokines IL-6 and IL-10, which, according to several studies, are predictive of poor outcome in ARDS, if elevated^23–25^. In this line of evidence, we report significantly reduced cytokine levels in the BALF in mice treated with F7 compared to mice treated with the control DARPin and PBS. As expected, the pro-inflammatory cytokines IL-1β, TNF-α, IFN-γ, IL-6 were significantly reduced in the F7 group underlining its strong anti-inflammatory effects. The decreased local inflammatory response may be one of the contributing factors to improved survival observed in the F7 group. We also found significantly reduced levels of IL-12 in the BALF in mice treated with F7. IL-12 promotes the release of vascular endothelial growth factor, which in turn increases vascular permeability^26,27^. By reducing IL-12 levels, F7 may therefore improve the endothelial barrier function leading to reduced pulmonary edema and an attenuated local inflammatory response. Lower MCP-1 levels in the BALF, which we report for the F7 group, have also been associated with a dampened local inflammatory response after treatment in mouse models of LPS-induced acute lung injury^28,29^.

In the clinic, not only the neutrophil count in the BALF, but also the neutrophil-to-lymphocyte ratio in peripheral blood is an important prognostic factor in patients with ARDS^30^. In this line of evidence, our study demonstrated a significant reduction in the percentage of neutrophils in the peripheral blood in mice treated with F7 compared to PBS or the control DARPin. Furthermore, this effect can be regarded as evidence of a reduced systemic inflammatory reaction in the F7 treated animals^31^.

The percentage of blood neutrophils is often increased in animals experiencing stress^31^. Consequently, the percentage of neutrophils was significantly increased in the blood of all LPS-treated mice compared to healthy mice. As we quantified leukocyte subpopulations as percentage of total leukocytes, the lower percentages of lymphocytes observed in the LPS treated groups compared to healthy controls most likely reflect the proportional increase of neutrophils in the LPS-treated groups and not lymphocyte migration.

In the chronic phase, the percentage of blood neutrophils was substantially, yet not significantly increased in mice treated with F7 compared to those treated with the control DARPin or PBS. Most likely, this finding is coincidental since the elevated mean percentage of neutrophils in the F7 group is strongly influenced by one mouse with a neutrophil percentage of 59% which is much higher than in the other F7 treated mice. However, this mouse was included in our analysis as it was not identified as an outlier using the statistics described in the “Methods” section.

To fulfil their central role in the development of ARDS, neutrophils need to be activated and migrate into the lung^18^. Mac-1 on the neutrophil surface is the main mediator of neutrophil migration during experimental LPS induced murine ARDS^32^. We previously demonstrated that DARPin F7 binds preferentially to activated neutrophils which are highly pro-inflammatory^14^. This characteristic of F7 has the potential to reduce inflammation in a highly specific manner while avoiding severe immunosuppression since only activated neutrophils are targeted. Furthermore, protein modifications (e.g. albumin binding domains, PEGylation) can be used to bioengineer the serum half-lives of DARPins from days to weeks enabling an effective period as needed and shortening the period of immunosuppression^33^.

Mac-1 has previously been targeted for therapeutic purposes in mouse models of acute lung injury^32,34^. Although local inflammation was reduced, survival data was not presented. Our data, however, shows that activation-specific anti-Mac-1 treatment with DARPin F7 not only reduces inflammation but also improves survival. Our results are likely to have been favored by the inherent properties of F7. For example, due to its smaller size compared to immunoglobulins (17 kD vs. approx. 150 kD) DARPins can penetrate deeper into tissue and local areas of inflammation. Additionally, DARPins are highly stable and can be produced and purified relatively easily at high yields leading to low costs^11^. Moreover, DARPins have been shown to be safe for systemic application in humans and phase II trials are currently ongoing investigating the effects of DARPin MP0250 against multiple myeloma^35^.

Conventional non-activation specific anti-integrin antibodies were used in previous animal studies of acute lung injury. However, non-activation-specific targeting of integrins has been associated with severe complications. For example, efalizumab, which targets the α_L_β_2_ integrin, was initially approved for the treatment of psoriasis but later removed from the market after multiple cases of progressive multifocal encephalopathy^36^. Another example is abciximab, which targets the α_IIb_β_3_ integrin, effectively inhibits platelet aggregation during percutaneous coronary interventions in cases of myocardial infarction. However, it is associated with severe thrombocytopenia and uncontrolled bleeding complications^37^. Studies have shown that, in the case of α_IIb_β_3_, effective antithrombotic treatment can be achieved while minimizing bleeding complications by targeting only the activated conformation of α_IIb_β_3_ using a single chain antibody^38^.

A mouse model of ARDS used to investigate novel treatment options should be as similar as possible to human ARDS. Matute-Bello et al. previously published an official workshop for the American Thoracic Society aiming to standardize read-outs for animal models of ARDS^16^. According to these suggestions, histological evidence of tissue injury, the alteration of the alveolar capillary barrier, and the inflammatory response in the BALF were determined as the three main features of experimental ALI. We followed these recommendations in analyzing histological sections of mice with experimental ALI. We used a well-established model of murine ARDS: intratracheal instillation of LPS. This model produces similar pathological characteristics as observed in human ARDS including microvascular and alveolar damage, intrapulmonary hemorrhage, edema and fibrin deposition^39^. These characteristic features were observed by animals which received LPS intratracheally but did not receive F7 i.p. indicating that ARDS was in fact induced. In accordance with the findings of Card et al., who demonstrated significant sex differences by the LPS induced murine ARDS, we only used male mice^40^.

Moreover, an advantage of our model is that it is robust and highly reproducible^39^. This is exemplified by the similar survival and body weight curves of the LPS-treated animals compared to previous studies using this model^41,42^. However, no murine model can completely mimic the complexity and various etiologies of human ARDS.

In this study we administered the first dose of DARPin F7 and control substances on the day of LPS instillation. Our data indicates that treating ARDS in its early stages before it reaches its peak severity might carry several advantages. In the clinic this is not impractical as it may take several days up to one week for ARDS to develop after an inciting event, for example, viral infection^1,43^. However, future studies will have to investigate whether delayed treatment with F7 is effective in established ARDS. This is not unlikely, since F7 targets activated neutrophils, and increased levels of neutrophils are reported in the BALF of patients with ARDS on day 7 and 14^44^.

This study is not without limitations. Recently, we demonstrated that DARPin F7 is an effective anti-inflammatory treatment in the cecal ligation and puncture sepsis model which is also a neutrophil driven disease^14^. Therefore, an anti-inflammatory effect was to be expected in the neutrophil driven model of acute lung injury. Moreover, detailed mechanisms of F7’s effects are not investigated in this study. As we did not analyze absolute leukocyte counts in whole blood and only present relative changes, we cannot with certainty infer how DARPin treatment affected absolute blood leukocyte blood counts.

## Conclusions

In conclusion, we demonstrate that early treatment with DARPin F7 inhibited neutrophil accumulation, reduced signs of local and systemic inflammation and improved survival in our murine model of ARDS. F7 may therefore be a novel anti-inflammatory drug candidate for the treatment of severe ARDS but future studies are required to determine its true potential.

## Methods

### Designed ankyrin repeat proteins (DARPins)

A DARPin named ‘E3_5’ was used as a control DARPin which does not bind to any known target and has previously been used as a control DARPin in animal experiments^45^. DARPin production and purification and testing were performed as described previously^14^. Expression vectors contained a T5 promotor enabling strong expression of DARPins in *E. coli* cells using Isopropyl-β-D-thiogalactopyranosid (IPTG). Furthermore, the vectors encode for ampicillin resistance, allowing selective growth in ampicillin-containing media. DARPins contained an albumin binding domain, which is leads to a DARPin half-life in vivo of approximately 1–2 days^33^. DARPins were produced in E. coli BL21 DE3 using LB medium containing ampicillin (F_c_ = 100 µg/ml). Expression was induced by IPTG when the OD_600_ reached 0.4–0.7 as determined by a spectrophotometer. After 5 h, cells were harvested by centrifugation and stored at -80 °C. Frozen bacterial pellets were lysed with Bugbuster Mastermix (Merck, Germany) following the manufacturer’s instructions and DARPins were allowed to bind to Ni–NTA agarose (Qiagen, Germany) for 1.5 h at RT. DARPins contained a 6 × His Tag allowing purification by Ni^+^-containing agarose beads. After incubation, Ni–NTA agarose was washed with buffers containing low concentrations of imidazole (10 and 20 mM). DARPins were then eluted with a buffer containing high concentrations (250 mM) of imidazole and dialyzed against PBS for at least 24 h to remove imidazole. Endotoxins were removed by phase separation using Triton X-114 (Sigma-Aldrich, USA) following standard protocols. Protein concentrations were determined using the Pierce™ BCA Protein Assay Kit (Thermo Fisher, USA). DARPin functionality was determined using flow cytometry.

### Mouse model of acute lung injury by endotracheal LPS instillation

8–10 weeks old male C57BL/6 J mice were purchased from Janvier Labs (Le Genest-Saint-Isle, France). All animals had free access to drinking water and food and were kept in the facilities of the Center for Experimental Models and Transgenic Service, Freiburg, Germany. All experiments were conducted strictly according to the German animal protection law and in accordance with good animal practice as defined by the Federation of Laboratory Animal Science Associations (www.felasa.eu) and the national animal welfare body GV-SOLAS (www.gv-solas.de). The study was carried out in compliance with the ARRIVE guidelines. The experimental protocols were approved by the responsible regional authorities (‘Tierversuchskomission Regierungspräsidium Freiburg’ 35-9185.81/G-19/122). Animals were divided into four groups (Fig. 1). Three groups received LPS intratracheally (ALI groups) while the healthy control group received PBS only.

LPS was purchased from Sigma-Aldrich (St. Louis, USA). The intratracheal instillation was performed under isoflurane anesthesia. Animals in the ALI groups received LPS (5 mg/kg) dissolved in 50 µl PBS intratracheally. Healthy control animals received the same volume of PBS only intratracheally. Animals were treated with 200 µg DARPin F7, control DARPin or PBS daily. Treatments were applied intraperitoneally (i.p.). The first application of DARPins or PBS was performed directly before the intratracheal LPS/PBS instillation (day 0). Animals were checked daily for signs of distress and weighed on day 0, 3, 6, 9 and before euthanasia. According to the protocol, mice were euthanized by exsanguination under ketamine (100 mg/kg)/xylazine (5 mg/kg) narcosis on day 4 for the acute phase groups and on day 10 for the chronic phase groups. Animals were euthanized before this time point if they lost more than 20% of their initial body weight or showed signs of extreme distress (Table 1). Mice surviving until day 4 were counted as survivors. Probability of survival was calculated by dividing the number of surviving mice by the number of total mice (acute and chronic phase) and multiplying the result with 100%.

### Bronchoalveolar lavage

Bronchioalveolar lavage (BALF) sampling was performed as described elsewhere^46^. Briefly, the trachea was visualized, and a 26 G catheter was inserted. The left main bronchus was ligated to preserve the lung for histological analysis. The right lung was filled with 0.5 ml sterile PBS three times and approximately a total of 0.6 ml BALF could be aspirated. Samples were centrifugated (2500*g*, 5 min) and the cell pellet was resuspended in 100 µl PBS. The resuspended pellet was analyzed by flow cytometry and the total protein concentration of the supernatant was determined using the Pierce™ BCA Protein Assay Kit.

### Flow cytometry

Leukocyte distribution and concentrations in the BALF were determined using multicolor flow cytometry. DARPins contain a His-tag allowing detection by a secondary Alexa Fluor 488 anti-His-tag antibody. The resuspended cell pellet was stained with antibodies solved in PBS supplemented with 0.5% bovine serum albumin (BSA). A master mix was prepared and 10 µl of the master mix was added per sample. The following antibodies were used to prepare the master mix: BV 510 anti-CD45.2, PE-Cy7 anti-CD19, PerCP-Cy5.5 anti-CD3, PE anti-Ly6G, APC anti-SiglecF (APC) and an Alexa Fluor 488 anti-His-tag antibody (all from Biolegend, USA), PE Texas Red anti-CD11b (Thermo Fisher, USA) and V450 anti-Ly6C (BD Biosciences, USA). Absolute cell counts in the BALF were determined using Trucount™ tubes (BD, USA) following the manufacturer’s instructions.

### Histology and lung injury scoring system

The left lung was filled with OCT compound diluted in PBS. After fast freezing, 8 µm thick tissue sections were prepared and stained with hematoxylin and eosin according to standard protocols. Histological scoring was performed in a blinded manner following the lung injury scoring system previously described by Matute-Bello et al.^16^ (Table 2). According to Matute-Bello et al., the main features of experimental ALI include histological evidence of tissue injury, alteration of the alveolar capillary barrier, presence of an inflammatory response, and evidence of physiological dysfunction. Ten fields of vision were analyzed. The number of neutrophils in the alveolar (A) and interstitial space (B), the amount of hyaline membranes (C) and proteinaceous debris (D), as well as the degree of alveolar septal thickening (E) were quantified and given individual scores (Table 2). The independent variables were weighted, and a lung injury score was calculated using the following formula: Score = [(20 × A) + (14 × B) + (7 × C) + (7 × D) + (2 × E)]/ (number of fields × 100).

**Table 2:** Histological scoring of lung sections.

Parameter	Score per field		
	0	1	2
A. Neutrophils in the alveolar space	None	1–5	> 5
B. Neutrophils in the interstitial space	None	1–5	> 5
C. Hyaline membranes	None	1	> 1
D. Proteinaceous debris filling the airspaces	None	1	> 1
E. Alveolar septal thickening	< 2x	2x–4x	> 4x

The score for each animal was calculated as follows = [(20 × A) + (14 × B) + (7 × C) + (7 × D) + (2 × E)]/(number of fields × 100).

### Quantitative real-time polymerase chain reaction (qRT-PCR)

RNA was extracted from frozen murine lungs using Qiazol and RNeasy Mini Kit (Qiagen, Valencia, CA, USA) according to the manufacturer’s instructions. Quantitative TaqMan-PCR was performed using a Bio-Rad CFX96 Touch Real-Time PCR System and TaqMan probes Mm00446190_m1 (IL6), Mm01288386_m1 (IL10). Data were analyzed using the 2^−∆∆Ct^ method. mRNA levels were normalized using GAPDH as a housekeeping gene and then standardized to the mean value of the respective healthy control group.

### Cytokine analysis

Cytokine levels in the BALF from the acute phase were analyzed flow-cytometrically using a commercially available bead-based kit (Legendplex™, Biolegend, USA) following the manufacturer’s instructions with the following exceptions. Samples were not-pre diluted, no Matrix-C was used and the incubation with beads was performed over night at 4 °C.

### Statistics

Data are presented as mean ± standard error of the mean (SEM). A Kolmogorov–Smirnov test was performed to test for normal or lognormal distribution. Data with lognormal distribution was log-transformed. Outliers were detected with ROUT method (Q = 1%). A two-tailed Student’s t-test was used when means of two groups were compared. Survival curves were compared by a log rank test. Statistics were performed using GraphPad Prism V9.0.1 software (GraphPad, USA). A *p* value ≤ 0.05 was considered statistically significant. *p < 0.05, **p < 0.01, ***p < 0.001.

### Ethics approval and consent to participate

All animal studies were approved by the responsible regional authorities (‘Regierungspräsidium Freiburg’).

## Supplementary Information

Supplementary Figures.

## Data Availability

The datasets generated and analyzed in this study are available from the corresponding author upon reasonable request.

## Abbreviations

ALI: Acute lung injury
APC: Allophycocyanin
ARDS: Acute respiratory distress syndrome
BALF: Bronchoalveolar lavage fluid
BCA: Bicinchoninic acid
BD: Becton Dickinson
BV: Brilliant Violet
COVID-19: Coronavirus disease 2019
DARPin: Designed ankyrin repeat protein
IPTG: Isopropyl β-d-1-thiogalactopyranoside
kD: Kilodalton
LPS: Lipopolysaccharide
OCT: Optimal cutting temperature
PBS: Phosphate buffered saline
PE: Phycoerithrin
PerCP: Peridinin chlorophyll
RT: Room temperature
SARS-CoV-2: Severe acute respiratory syndrome coronavirus type 2
SD: Standard deviation
SEM: Standard error of the mean
USA: United States of America
