# Supplementary material for: An activation specific anti-Mac-1 designed ankyrin repeat protein improves survival in a mouse model of acute lung injury
Source: Sci Rep. 2022 Apr 15;12:6296. doi: 10.1038/s41598-022-10090-6 (PMC9012056; doi:10.1038/s41598-022-10090-6)
Supplement: Supplementary file 1 — Supplementary Figures. [file 41598_2022_10090_MOESM1_ESM.pdf]

An activation specific anti-Mac-1 Designed Ankyrin Repeat Protein improves survival in a mouse model of acute lung injury

Patrick M Siegel, MD[1]; Anne-Sophie Przewosnik[1]; Jan Wrobel[1]; Timo Heidt[1], MD; Martin Moser, MD[1]; Karlheinz Peter, MD, PhD[2,3,4]; Christoph Bode, MD[1]; Philipp Diehl, MD, PhD[1†], István Bojti, MD[1*†];

[1] Department of Cardiology and Angiology I, University Heart Center Freiburg – Bad Krozingen, Faculty of Medicine, University of Freiburg, Freiburg, Germany

[2] Atherothrombosis and Vascular Biology Laboratory, Baker Heart and Diabetes Institute, Melbourne, Australia

[3] Department of Medicine, Central Clinical School, Monash University, Melbourne, Australia

[4] Baker Department of Cardiometabolic Health, University of Melbourne, Melbourne, Australia

† István Bojti and Philipp Diehl contributed equally to this article.

*Corresponding author & address for reprints

Dr. Istvan Bojti, Department of Cardiology and Angiology I, Heart Center Freiburg University, Faculty of Medicine, University of Freiburg, Hugstetter Str. 55, Freiburg, 79106, Germany, Tel: +49 761/270-34010 E-mail: istvan.bojti@uniklinik-freiburg.de

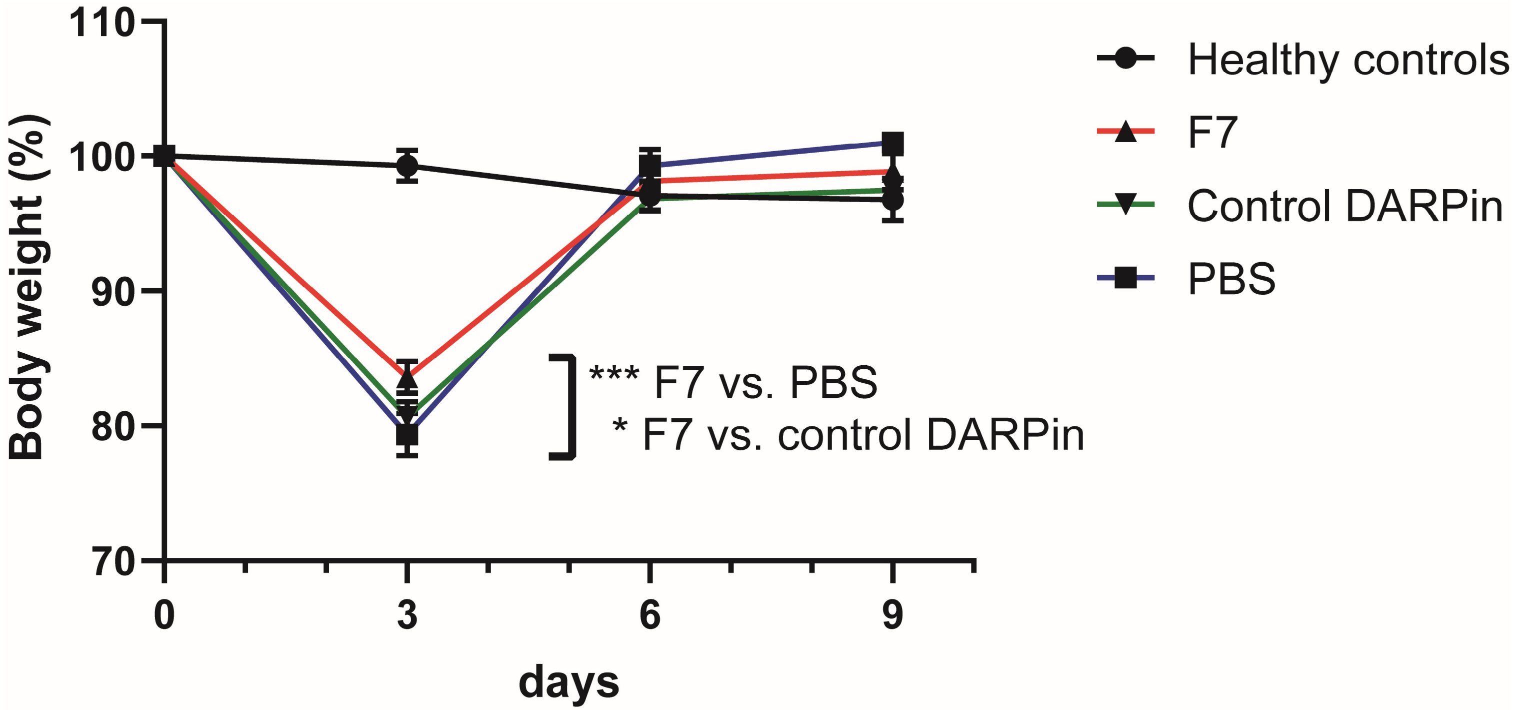

Supplementary Figure S1. Mean weight (in percentage of starting weight on day 0) over time during the acute and chronic phase of the mouse model of acute lung injury. Mice treated with the control DARPin or PBS suffered more severe weight loss compared to mice treated with F7, particularly during the acute phase, as exemplified on day 3. Data are presented as mean±SEM. n=10-35 mice per treatment group, *p<0.05, ***p<0.001, Body weight on day 3 was compared using an unpaired t-test.

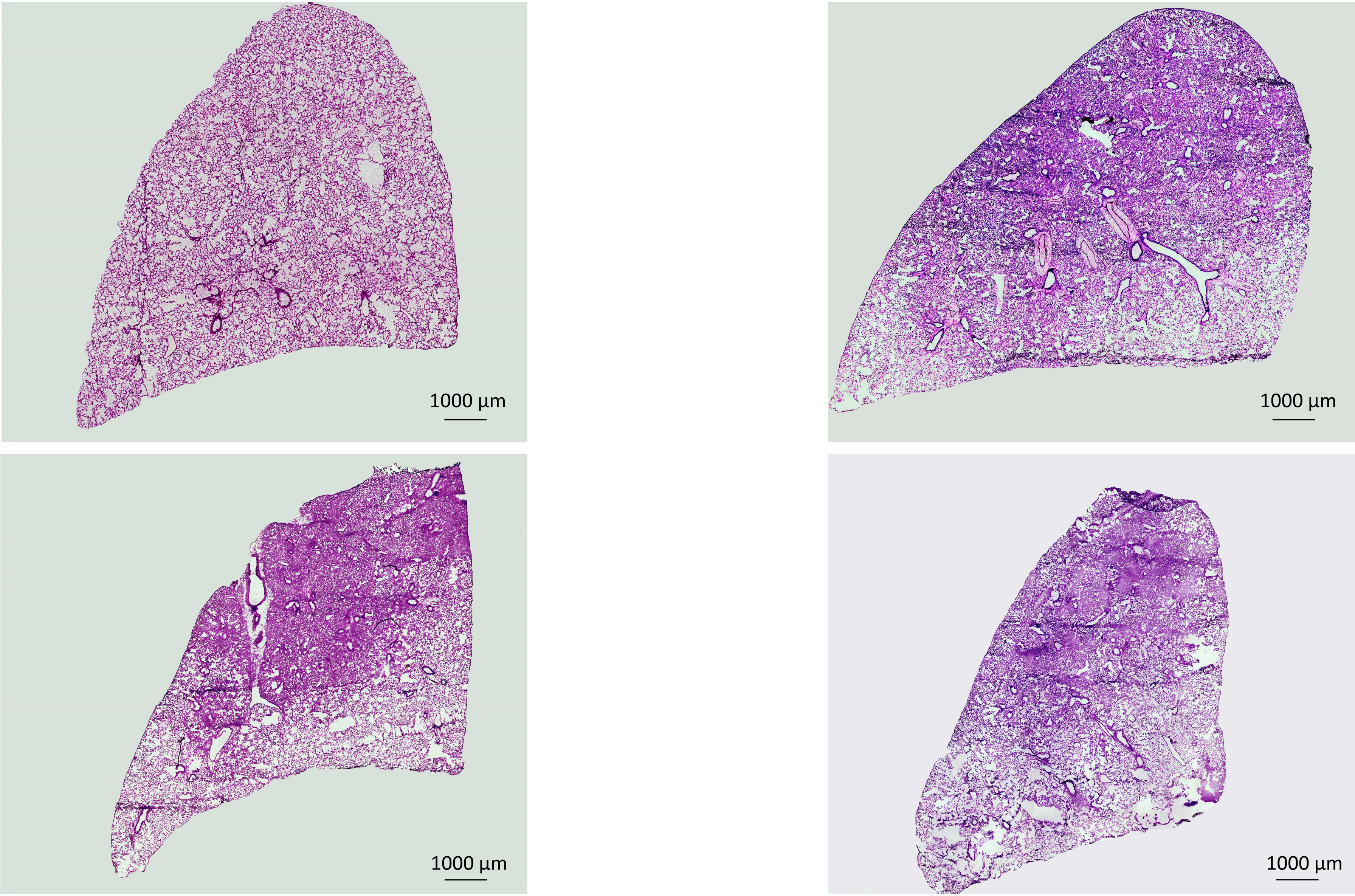

Supplementary Figure S2. Representative low power field whole lung images of the treatment groups. (a) healthy control (b) PBS (c) control DAPRin (d) DARPin F7.

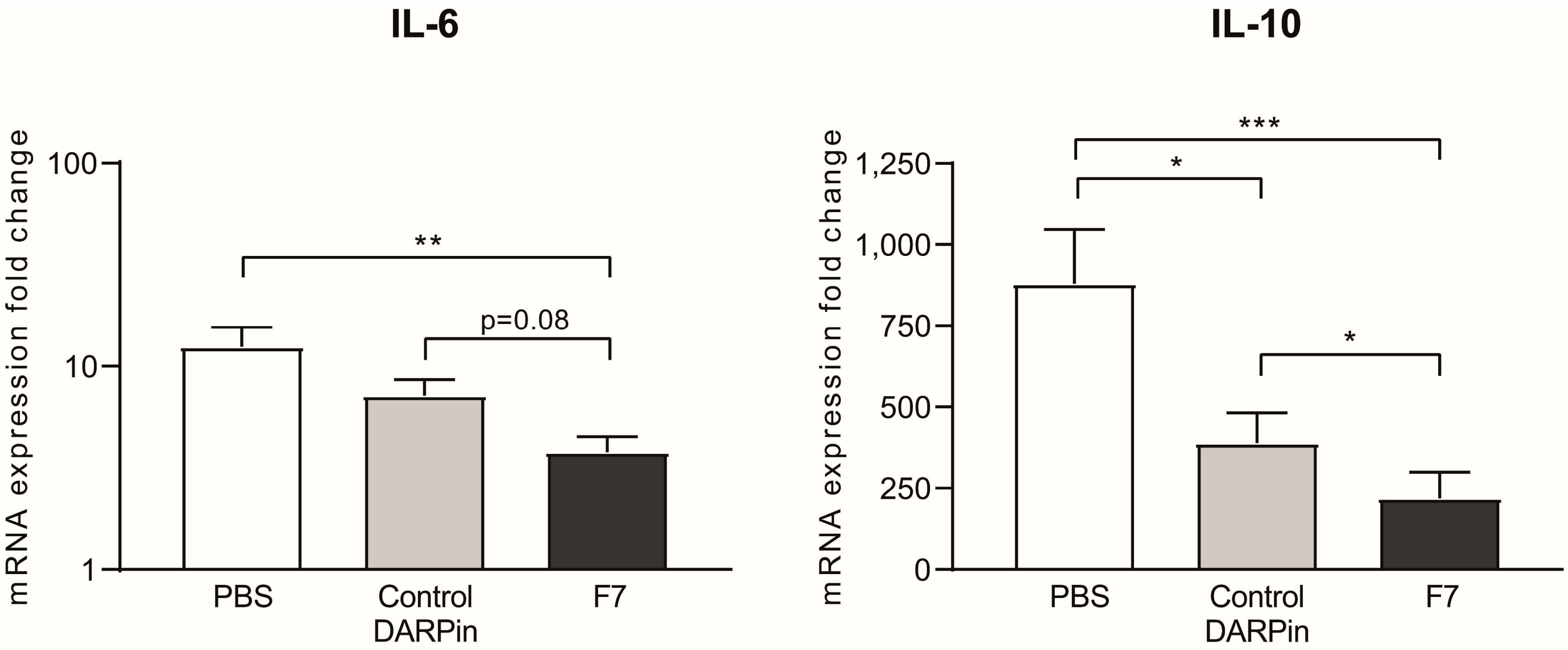

Supplementary Figure S3. mRNA expression of cytokines IL-6 and IL-10 in lungs of LPS-treated mice. Experiments were carried out and data were analysed as described in the Methods section. Data are presented as mean±SEM. n=11-18 mice per treatment group. *p<0.05, **p<0.01, ***p<0.001. Treatment groups were compared by an unpaired t-test.

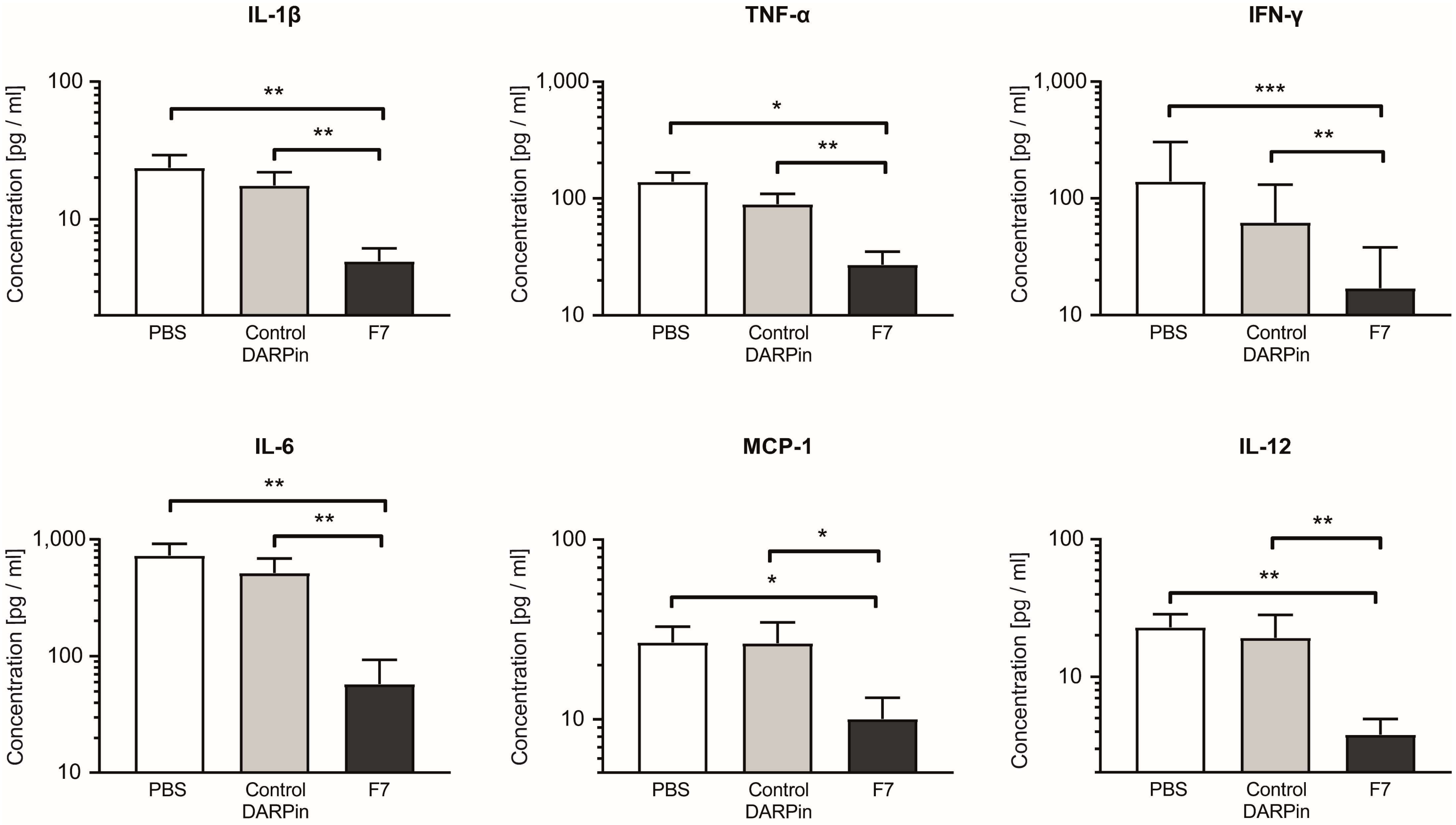

Supplementary Figure S4. Cytokine levels in the bronchoalveolar lavage (BALF) in mice receiving with intratracheal LPS on during the acute phase. A Mice were treated either with PBS, the control DARPin or DARPin F7. Cytokine analysis in the BALF was carried out as described following the manufacturer's protocol using a commercially available bead-based cytokine array (Legendplex^{TM}, Biolegend, USA). Data showed a log-normal distribution and are presented as mean±SEM. n=11-18 mice per treatment group. *p<0.05, **p<0.01, ***p<0.001. Treatment groups were compared by an unpaired t-test.
